# Infected “Mycotic” Aneurysm of the Common Carotid Artery—A Differential Diagnosis to Tumor of the Neck

**DOI:** 10.3389/fsurg.2018.00075

**Published:** 2018-12-10

**Authors:** Patrick R. G. Eriksen, Gitte B. Hvilsom, Preben Homøe

**Affiliations:** Department of Otorhinolaryngology and Maxillofacial Surgery, Zealand University Hospital, Køge, Denmark

**Keywords:** mycotic aneurysm, unknown primary cancer, differentional diagnostic, diagnostic imaging, neck surgery, syphilis, deep neck infections

## Abstract

**Introduction:** Infected “mycotic” Aneurysm (IA) of the extracranial carotid artery is a rare condition that can be fatal if mistaken for other pathology. An 83-year-old man presented with a mass on the neck initially suspected malignant. Weeks later it grew rapidly and was found to be an IA, thus requiring acute surgery. Via this case report, we discuss diagnostics and approach when diagnosing masses in relation to vessels of the neck not readily explained.

**Case Report:** After diagnostic imaging and clinical assessment an unknown primary tumor of the neck was suspected. Fine needle aspiration was inconclusive. The patient did not present with any signs of infection or neurological symptoms—only discomfort and pain. Approximately two weeks later, the mass grew and the patient became dysphagic, febrile, and confused. Computed tomography angiography revealed an IA of the right common carotid artery. The patient underwent acute surgery consisting of ligation of the internal and external carotid arteries and resection of the internal jugular vein. The pathogen found was *E. coli*, supposedly from the bladder after surgical intervention due to polyposis.

**Conclusion:** IA is a very rare entity and can have many etiologies. Since it can be fatal, it is necessary to keep IA in mind when diagnosing masses in relation to vessels of the neck. As shown in this case of a *E. coli*-induced IA, patients can present with atypical symptoms, on diagnostic imaging it can be mistaken for other pathology, and pathogenesis can be unclear.

## Introduction

Masses of the neck can be caused by immensely diverse pathologies. Among these are: lymphadenopathy (e.g., cat scratch fever, tuberculosis, toxoplasmosis), congenital neck lesions (e.g., thyroglossal duct cyst, branchial cleft cyst), and persistent masses with high potential for malignancy either as a primary tumor or metastases from Unknown Primary Tumor (UPT) (1).

At the beginning of 2008, a national fast-track program for head and neck cancer was introduced in Denmark. The goal was to improve survival by reducing the waiting time from initial suspicion of malignancy to the start of treatment (2). When specialist practitioners suspect malignant disease of the head and neck the patient is examined as fast as possible within six working days at an Ear, Nose, and Throat (ENT) department. If suspicion of cancer is upheld, the patient will be sent to relevant diagnostic imaging within days.

We have chosen to submit the following case study of an elderly man who was seen in the department's fast-track program with a tentative diagnosis of UPT of the neck. Diagnostic imaging supported this diagnosis, and further attempts of histological specification were undertaken without conclusive results. Furthermore, the PET/CT showed uptake both in the lungs and the colon, thus postponing ENT-diagnostics due to colonoscopy and planned bronchoscopy. Approximately 2 weeks after the initial visit, the tumor grew rapidly in size and was diagnosed via a Computed Tomography (CT)-angiography as an Infected “mycotic” Aneurysm (IA)—an extremely rare condition especially in the Common Carotid Artery (CCA) (3).

## Case Report

An 83-year-old man was referred from an ENT specialist practitioner to the ENT-department due to a painful process situated in level II-III on the right side of the neck. He had a history of arterial hypertension, low-malignant carcinoma *in situ* of the bladder for which we had undergone a transurethral resection whereafter he developed postoperative sepsis, and one perioperative episode of arrhythmia with consequent cardiac arrest during hip surgery in 2017. The process had developed over ~3 weeks. The patient had experienced pain and discomfort with right-sided otalgia upon swallowing. He had no signs of dysphonia, was afebrile, had no erythema or calor in relation to the mass, and no neurological abnormalities. Thus, infection was not a provisional diagnosis. Ultrasound showed a hypoechoic, ill-defined tumor mass sheathing ~50 percent of the right CCA. No flow in the mass was detected with Color Doppler Ultrasound. Therefore, UPT was suspected.

Fine needle aspiration was inconclusive twice. Open biopsy and core biopsy was not an option due to the uncertainty of malignancy and potential spreading and bleeding. Therefore, diagnostic imaging was ordered.

**Figure 1:**
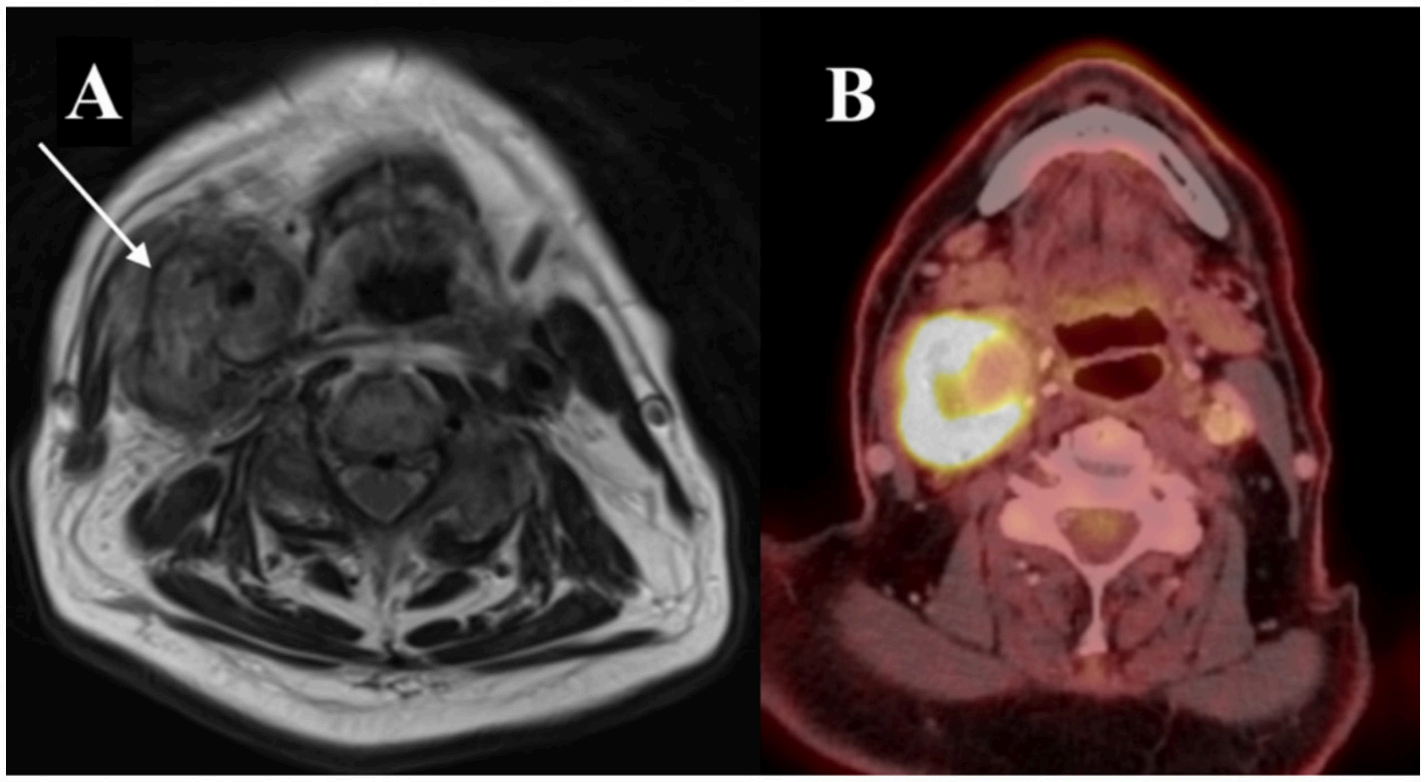
Initial diagnostic imaging. **(A)** MRI of the neck in the transverse plane. Tumor marked with an arrow. **(B)** PET/CT in the transverse plane showing uptake in relation to a slightly dilated right CCA.

Magnetic Resonance Imaging (MRI) and Positron Emission Tomography/Computed Tomography (PET/CT) showed what was concluded to be a tumor suspected of malignancy sheathing the carotid artery, though a slight dilation of ~2.3 cm of the right CCA was detected (Figure 1). Furthermore, the PET/CT showed uptake both in the lungs and the colon, thus postponing further ENT-diagnostics due to the search for the primary focus of the tumor. Since malignancy/metastasis was suspected, no initial blood tests for infection were conducted.

Sixteen days after being enrolled in the ENT fast-track program, having undergone a colonoscopy and initial examination at the department of pulmonary medicine, the patient was admitted due to pain and further growth of the process on the neck. The patient was not able to eat or drink sufficiently and was experiencing general fatigue. The patient's family found that the patient's mental condition had deteriorated.

Upon admission, his vital signs were: A temperature of 38.4°C, 172/98 mm Hg blood pressure, a heart rate of 105 beats/min, and an unlabored respiratory rate of 16/min.

Biochemistry showed markedly high inflammatory indices with a c-reactive protein of 266 mg/L, white blood cell count of 26.9 × 10^9^/L, and 24.1 × 10^9^/L neutrophils. A urine sample was sent to the laboratory for cultivation and analysis of sensitivity.

**Figure 2:**
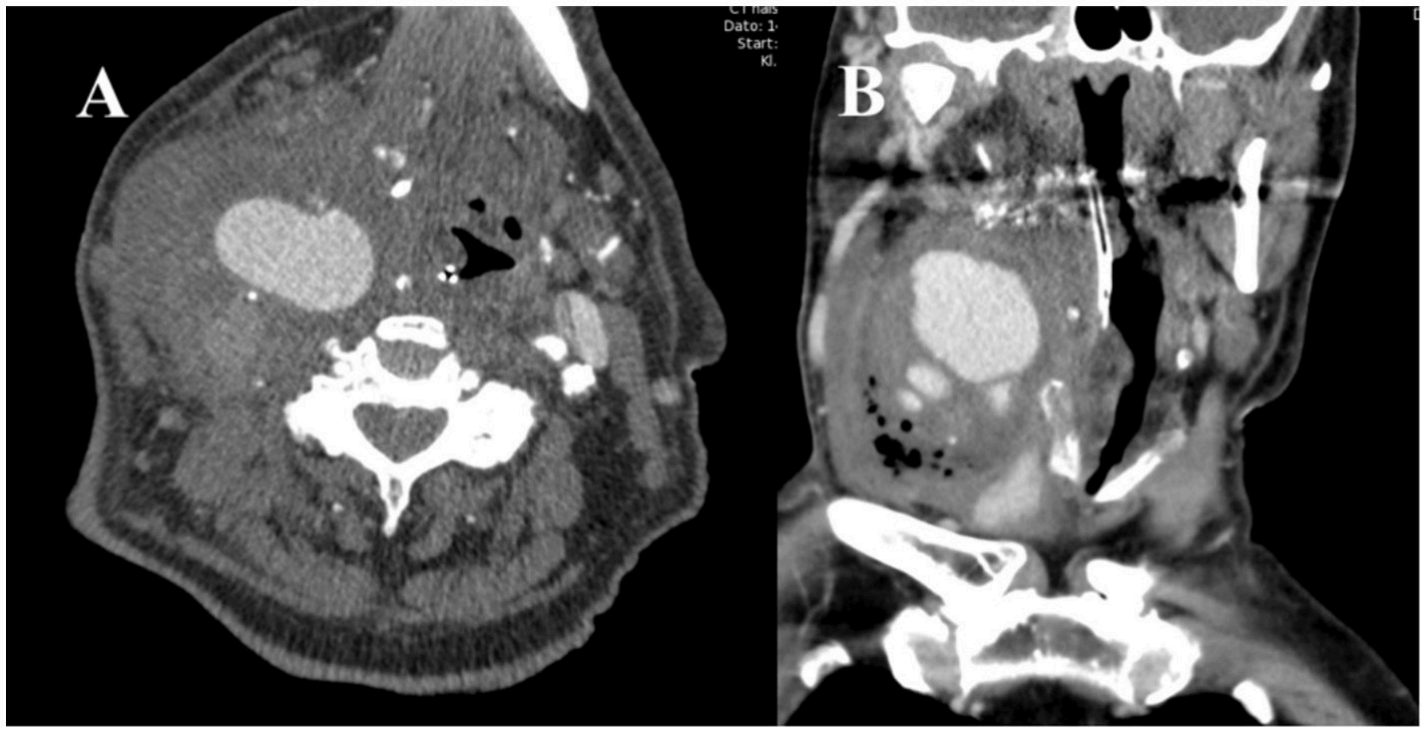
CT-angiography showing contrast in a now dilated, growing IA. **(A)** Transverse plane **(B)** Coronal plane.

Initially, the patient was prescribed Piperacillin and Tazobactam, treating symptoms as an infection with unknown primary focus. A diagnostic ultrasound was performed, which raised the suspicion of an aneurysm. CT-angiography showed an IA of about 5.4 × 3.9 cm (Figure 2). The patient was transferred to the vascular surgery department. Surgery consisted of resection of an 8.0 × 5.0 cm IA. Because of massive inflammation of the area involving both the external and the internal carotid artery and thrombosis of the internal carotid artery, both arteries were ligated and oversewn. Furthermore, due to thrombosis and necrosis, the internal right jugular vein was resected. A culture from the surgical site was positive for *E. coli* and relevant antibiotics were administered.

The only sequela was dysphonia due to paralysis of the right recurrent nerve.

After the resection of the IA, growth of *E. coli* with the same resistance pattern as seen in the IA was found in the urine samples from the admission date. This suggested the bladder to be the primary focus of infection.

## Discussion

First described by Sir William Osler in 1885, the term “mycotic” relates to the initial description of aneurysms with fungal appearance seen in several cases of bacterial endocarditis (4). This makes “mycotic” somewhat of a misnomer, since both mycotic- and bacterial infection can give rise to a mycotic aneurysm, and the latter is by far the most dominant form (5). Therefore, the term Infected Aneurysm (IA) seems more appropriate. Being rare, the actual incidence of IA is uncertain. In 2013 Pirvu et al. found a total of 100 reported cases of Extracranial Carotid Artery (ECA) IAs with a relatively stable incidence of ~20 cases every decade (5). In contrast, the incidence of UPT in Denmark is much more common with 0.34 cases per 100,000 individuals, corresponding to ~20 cases per year (6).

Symptoms of an infected aneurysm of the ECA can vary depending on the size and position. These include the following symptoms: a growing, pulsatile cervical mass associated with pain, locally or radiating to the ear, tenderness, fever, dysphonia, dyspnea, and dysphagia (7, 8). Furthermore, cranial- and sympathetic-nerve involvement can lead to Horner's syndrome or parasympathetic-, sensory-, and/or motor dysfunction (7, 9). Untreated, a high percentage of cases will have a fatal outcome due to rupture and hemorrhage. Furthermore, septic embolization can occur, with risk of neurological sequelae due to arterial occlusion and associated emboli (10–12).

Treatment options include total resection, endovascular stenting, and autologous interposition vein graft which is the preferred contemporary method due to fewer complications (5, 13–15). In our case, a total resection was chosen due to the condition of the tissue and thrombosis. Though speculative, in the presented case an autologous vein graft would presumably have been an option if the aneurysm had been detected at an earlier stage, consequently reducing the overall risk for the patient.

Our case has several unique clinically interesting characteristics. The aneurysm was initially not pulsatile, there were no neurological symptoms, and the patient did not have any initial signs of infection; thus the primary diagnosis was UPT. PET/CT and MRI were performed as defined in the Danish fast-track program of head and neck cancers (2). Imaging described an unspecific tumorous mass and the tentative diagnosis was upheld. This, along with findings of suspicious uptake on PET/CT in the colon and lungs impaired the uncovering of the IA.

An IA is characterized by a positive culture result or evidence of other organisms on histological examination of the arterial wall (5). Only seven cases of *E. Coli*-induced IA in the ECA has been reported since 1966 (5, 16, 17). There are several hypotheses regarding the pathophysiological genesis of IAs of the CCA. These include iatrogenic vascular trauma (18) and septic emboli that carry the pathogen primarily to the bifurcation of the CCA, likely the pathogenesis in the present case (19). Aside from a distal origin, local infection (e.g., deep neck infections, cervical lymphadenitis), and periarterial lymphatics can spread the infection to the vessels (9). This is especially important to remember when examining pediatric patients, as this is the most common cause of extracranial carotid artery aneurysms in children. This is due to a higher incidence of lymphadenitis based on pharyngeal infections (e.g., tonsillitis, pharyngitis) (9, 20). Furthermore, IA has been reported in relation to Lemierre's syndrome both due to *Fusobacterium* and methicillin-resistant *staphylococcus aureus* (21).

Nowadays, the most prevalent pathogens are *Staphylococcus aureus, Salmonella*, and *streptococcus* species (5, 22). Previously, before the invention of antibiotics, syphilis and untreated endocarditis were the main causes of arteritis and subsequent IAs (23). Thought all but eliminated, the last two decades have shown a marked rise in cases of syphilis in the Western world, which could lead to new cases of syphilis-induced IAs (24–26). In regards to *E. coli*-induced IA, in this case the strain was not beta-lactamase-producing, but it has been argued that further cases may be seen in the future due to drug-resistant *E. coli* urinary-tract infections (13).

A history of sepsis, surgery, signs of pharyngitis/tonsillitis, trauma or procedures involving vascular puncturing of vessels of the neck support the diagnosis of IA (5). Even though IA of the ECA is a rare disease, it is an important differential diagnosis when findings suggest a close relation of a suspected tumor to arteries of the neck. To avoid delay of diagnosis and intervention, contrary to the presented case, if the evaluation of prior risks suggests, prompt vascular imaging should be considered. CT-Angiography is the modality of choice when diagnosing IAs, where information on the circumference, morphology, and state of contralateral vessels is essential for the evaluation of treatment options and prognosis (27, 28).

In conclusion, clinicians should think of IA as a possible etiology when diagnosing tumors of the neck in close proximity to the vessels. As discussed, both the presentation of IA and etiology can vary considerably, and should therefore always be considered. Consequently, a broad anamnesis of prior risk factors is very important for the detection. Early detection of the disease is imperative; though rare, an aneurysm of the carotid artery can have a fatal outcome. Furthermore, early detection can have relevance for surgical options and later complications.

## Ethics Statement

Written informed consent was obtained from the patient specifically for publication of this case report.

## Author Contributions

PE: General script, gathering information, photoshop, and consent from patient; GH: Specialist consulting, re-writing, reference search, and revisions; PH: Re-writing and revisions, reference search, and help with submission.

### Conflict of Interest Statement

The authors declare that the research was conducted in the absence of any commercial or financial relationships that could be construed as a potential conflict of interest.
